# Direct comparison of an ultrasensitive real-time PCR assay with droplet digital PCR for the detection of *PIK3CA* hotspot mutations in primary tumors, plasma cell-free DNA and paired CTC-derived gDNAs

**DOI:** 10.3389/fonc.2024.1435559

**Published:** 2024-12-06

**Authors:** Stavroula Smilkou, Loukas Kaklamanis, Ioanna Balgouranidou, Helena Linardou, Alkistis Maria Papatheodoridi, Flora Zagouri, Evangelia Razis, Stylianos Kakolyris, Amanda Psyrri, Christos Papadimitriou, Athina Markou, Evi Lianidou

**Affiliations:** ^1^ Analysis of Circulating Tumor Cells, Laboratory of Analytical Chemistry, Department of Chemistry, University of Athens, Athens, Greece; ^2^ Department of Pathology, Onassis Cardiac Surgery Center, Athens, Greece; ^3^ Department of Medical Oncology, University General Hospital of Alexandroupolis, Alexandroupolis, Greece; ^4^ Oncology Unit, Metropolitan Hospital, Athens, Greece; ^5^ Department of Clinical Therapeutics, School of Medicine, Alexandra Hospital, National and Kapodistrian University of Athens, Athens, Greece; ^6^ Third Department of Medical Oncology, Hygeia Hospital, Athens, Greece; ^7^ Section of Medical Oncology, Department of Internal Medicine, Faculty of Medicine, Attikon University Hospital, Athens, Greece; ^8^ Oncology Unit, 2nd Department of Surgery, School of Medicine, Aretaieio Hospital, National and Kapodistrian University of Athens, Athens, Greece

**Keywords:** *PIK3CA* mutations, liquid biopsy, breast cancer, plasma cell-free DNA, circulating tumor cells, DNA melting curve analysis, ultrasensitive molecular assay, droplet digital PCR

## Abstract

**Introduction:**

Detection of *PIK3CA* mutations in primary tumors and liquid biopsy samples is of increasing importance for treatment decisions and therapy resistance in many types of cancer. The aim of the present study was to directly compare the efficacy of a relatively inexpensive ultrasensitive real-time PCR with the well-established and highly sensitive technology of ddPCR for the detection of the three most common hotspot mutations of *PIK3CA*, in exons 9 and 20, that are all of clinical importance in various types of cancer.

**Patients and methods:**

We analyzed 42 gDNAs from primary tumors (FFPEs), 29 plasma-cfDNA samples, and 29 paired CTC-derived gDNAs, all from patients with ER+ metastatic breast cancer, and plasma from 10 healthy donors. The same blood draws were used for CTC isolation using EpCAM beads for positive immunomagnetic enrichment. All FFPEs and plasma-cfDNA samples were analyzed in parallel for *PIK3CA* mutations by ultrasensitive real-time PCR assay and droplet digital PCR.

**Results:**

In gDNAs from FFPEs, using ultrasensitive real-time PCR, the p.E545K mutation was detected in 22/42(52.4%), and the p.E542K and p.H1047R mutations were detected in 14/42(33.3%) and 16/42(38.1%), respectively. Using ddPCR, the p.E545K mutation was detected in 22/42(52.4%), p.E542K in 17/42(40.5%), and p.H1047R in 19/42(45.2%) samples, revealing a concordance between the two methodologies of 81%, 78.6% and 78.6% for each mutation respectively. In plasma-cfDNA, using ultrasensitive real-time PCR, the p.E545K mutation was detected in 11/29(38%) and both p.E542K and p.H1047R mutations in 2/29(6.9%).In the same plasma-cfDNA samples using ddPCR, p.E545K was detected in 1/29(3.5%), p.E542K in 2/29(6.9%), and p.H1047R in 3/29(10.5%) samples, revealing a concordance of 65.5%,100% and 93.1% for each mutation respectively. In paired CTC-derived gDNAs p.E545K was detected in 11/29(38%), p.E542K in 3/29(10.3%), and p.H1047R in 7/29(24.1%) samples.

**Conclusions:**

This low-cost, high-throughput and ultrasensitive real-time PCR assay provides accurate and specific detection of *PIK3CA* hotspot mutations in liquid biopsy samples and gives similar results to ddPCR. This assay can be performed in labs where digital PCR instrumentation is not available. In CTC-derived gDNA and paired plasma-cfDNA, *PIK3CA* mutations detected were not identical, revealing that CTC and plasma-cfDNA give complementary information.

## 1 Introduction

PI3K (Phosphoinositide 3-kinase) signaling is deregulated in a variety of cancers (1–4). The three main *PIK3CA* hotspot mutations, exon 9 p.E545K and p.E542K and exon 20 p.H1047R, are detected approximately in 40% of hormone receptor positive (HR+) breast cancer, mainly in the helical and kinase domains of the *PIK3CA* gene (5, 6). Beyond breast cancer, *PIK3CA* mutation detection is also very important in other types of cancer like lung (7), colorectal (4, 8), anal squamous cell carcinoma (9) and pancreatic cancer (10).

In HR+ metastatic breast cancer patients with lack of human epidermal growth factor receptor-2 (HER2) overexpression and/or amplification, the widely accepted therapy is the combination of endocrine therapy (ET) with cyclin-dependent kinases (CDK)4/6 inhibitors (11). Therapeutics targeting specific driver mutations in *PIK3CA* have revolutionized the treatment of breast cancer in recent years (6, 12, 13). Alpelisib, a PIK3 inhibitor, has been approved by both the U.S. Food and Drug Administration (FDA) and the European Medicines Agency (EMA) for use in combination with fulvestrantin patients with HR+, HER2-negative (HER2-), *PIK3CA*-mutated, advanced or metastatic breast cancer following progression on or after treatment with an endocrine-based regimen (14–17). Moreover, triplet therapy with palbociclib, taselisib, and fulvestrant has been reported to have a positive effect in patients with heavily pretreated *PIK3CA*-mutantER-positive (ER+)/HER2- advanced breast cancer (18). There are several factors that ultimately result in resistance to PI3K inhibitors such as a) inactivation or loss of phosphatase and tensin homolog (*PTEN)* activity, b) mutations and amplification of PI3K, c) drug-related toxicities and d) various resistance mechanisms (19).

Liquid Biopsy analysis (LBx), is minimally invasive and enables longitudinal follow-up for cancer patients in real time (20–22) through the analysis of circulating tumor cells (CTCs), plasma-cell-free DNA (cfDNA) and circulating tumor DNA (ctDNA (22, 23). LBx is very important for predicting disease progression or relapse, as well as monitoring response to treatment in breast cancer (24, 25), and minimal residual disease (MRD) detection (26–28). It is highly important to note that the implementation of LBx in clinical practice requires extensive standardization and analytical validation of protocols in every step of processing (24). There is now a variety of commercially available assays for *PIK3CA* mutation detection mainly based on PCR-based and next-generation sequencing (NGS) methodologies (29–34). Detection of *PIK3CA* mutations in CTCs has been reported in a few studies so far (35–39). This is a very challenging and demanding procedure, since CTC are usually detected at very low numbers in circulation, and moreover are highly heterogeneous (21–23). Detection of *PIK3CA* mutations in plasma-cfDNA has shown that ctDNA analysis can capture most of the mutations found in tissue biopsy, and the level of concordance ranges from 72.5% to 100%, depending on the different techniques used and the timing of tissue biopsy (37).

A plethora of research papers indicate that CTCs and plasma-cfDNA are complementary and both should be evaluated in parallel to gain more detailed information on the mutational landscape of a patient’s tumor. It was reported that a combination of CTCs and ctDNA evaluation for *PIK3CA* mutations in colorectal cancer is necessary in the clinical setting to achieve optimal surveillance of the course of disease and selection of the appropriate treatment (40). Another study comparing *PIK3CA* mutations in CTCs and primary tumors, revealed that CTCs can exhibit heterogeneity within a single patient, and may acquire additional genomic characteristics that differ from those of the primary tumor (36). Thus, a comprehensive liquid biopsy analysis that incorporates information from both ctDNA and CTCs, can be crucial for the selection of the appropriate treatment (37, 41–43). A relatively recent pilot study investigating *PIK3CA* mutations in ctDNA, CTCs, and extracellular vesicles (EVs), found consistent mutational profiles of EVs with CTCs, suggesting that EVs may have been released by CTCs (44).

Our team has reported the development of methodologies for the detection of hotspot mutations in exons 9 and 20 of the *PIK3CA* gene in primary tumors (45), and CTCs and ctDNA based on the combination of allele-specific priming, asymmetric PCR (ARMS-PCR), and melting curve analysis (35). Using this assay we analyzed plasma-cfDNA and paired CTCs as well as gDNAs isolated from CellSearch^®^ cartridges for two hotspot *PIK3CA* mutations (p.E545K and p.H1047R) (38). We further developed highly sensitive methodologies for the detection of *PIK3CA* hotspot mutations based on Nuclease-Assisted Minor Allele Enrichment Using Overlapping Probes-Assisted Amplification-Refractory Mutation System (NAPA assay) (46) and a dual-Drop-off droplet digital PCR (ddPCR) Assay for the simultaneous detection of ten hotspot *PIK3CA* mutations (47).

The aim of the present study was to directly compare our previously described ultrasensitive real-time PCR assay (35) for the detection of three hotspot *PIK3CA* mutations with ddPCR in primary tumors and plasma-cfDNA, and directly compare the *PIK3CA* mutational status in CTC-derived gDNAs and paired plasma-cfDNA samples.

## 2 Materials and methods

### 2.1 Sample collection and processing

Forty-two FFPE samples from ER+/HER2- metastatic breast cancer patients, collected in the period of 2020-2022, were obtained from the Department of Pathology, Onassis Cardiac Surgery Center. gDNA was isolated from FFPEs using the QIAamp DNA FFPE Tissue Kit (Qiagen, Hilden, Germany), according to the manufacturer’s instructions. In parallel, peripheral blood (PB) in EDTA (10mL) was prospectively collected from 29 patients with ER+/HER2-metastatic breast cancer and 10 healthy donors. PB samples were collected in participating clinical centers under the Operational Program Competitiveness, Entrepreneurship and Innovation, under the call RESEARCH-CREATE-INNOVATE (project code: T1RCI-02935). All patients gave their informed consent and the study was approved by the Ethic committees from all participating institutions. Plasma was obtained by two consecutive centrifugations (530xg for 10min at room temperature followed by a second centrifugation at 2000xg for 10min) within 2-4h and was further stored at −70°C until analyzed. Plasma-cfDNA was extracted from 2mL of plasma using the QIAamp DSP cNA Kit (Qiagen, Hilden, Germany), and cfDNA was eluted in 30μL elution buffer (38). CTC-derived gDNA was obtained from identical blood draws as previously described (39). gDNA from CTCs was isolated using the QIAamp DNA Micro Kit, Qiagen (Qiagen, Hilden, Germany), according to the manufacturer’s instructions. DNA quantification in all samples was performed using the NanoDrop™ 1000 Spectrophotometer (ThermoFisher Scientific). All experimental procedures were performed in different rooms, dedicated labware and areas to avoid contamination. All preparation steps for the ddPCR setup were performed in a dedicated pre-PCR room and a PCR hood dedicated for the preparation of ddPCR reactions.

### 2.2 *PIK3CA* mutation analysis using the ultrasensitive real-time PCR assay

The ultrasensitive real-time PCR *PIK3CA* assay detects three *PIK3CA* hotspot mutations (exon 9 p.E542K, p.E545K and exon 20 p.H1047R) as single-plex assays. The ultra sensitive real-time PCR assay is based on a combination of allele-specific, asymmetric rapid PCR (ARMS-PCR) and melting curve analysis. The analytical validation of the assay for two of these mutations (p.E545K and p.H1047R) was previously performed in detail reporting a limit of detection (LOD) of 0.05% (35). The assay was further extended to include the *PIK3CA* exon 9 p.E542K mutation and was analytically validated in the same way showing a similar LOD in the LightCycler^®^ 2.0 (IVD instrument, Roche Diagnostics, Germany) (results not shown).The interpretation of the results is performed by melting curve analysis as previously reported; the melting probe specific for each individual mutation provides a different melting temperature for its binding to the mutant allele as compared to its binding to the WT allele. PCR conditions and melting analysis protocols for each exon are described in detail in (35). Up to 50 ng/PCR DNA input is used. All samples were analyzed in the LightCycler^®^ 2. Primers and probes sequences are given in detail in Supplementary Table S3 (35).

### 2.3 *PIK3CA* mutation analysis using droplet digital PCR

Droplet digital PCR was performed in the QX200 AutoDG ddPCR System (Bio-Rad, USA). Using ddPCR all 42 FFPEs and 29 plasma-cfDNA samples were analyzed for the same *PIK3CA* mutations (E545K, E542K and H1047R). We used PrimePCR™ ddPCR™ Mutation Detection Assay Kits: *PIK3CA* WT for p.E542K, p.E545K, p.H1047R and *PIK3CA* p.E542K (Bio-Rad: #1863131), *PIK3CA* p.E545K (Bio-Rad: #1863132), *PIK3CA* p.H1047R, Human (Bio-Rad: #1863133), according to the manufacturer’s instructions. The LOD for each mutation using ddPCR is ~0.1%. We set the cut-off depending on the number of positive droplets that were detected in the healthy donors control group in each mutation channel. For *PIK3CA* p.E545K and p.H1047R the cut-off was 2 positive droplets (0.4 cop/μL) while for p.E542K this cut-off was 3 positive droplets (0.2 cop/μL). PCR of 10 ng/μl DNA input in all cases was performed in the C1000™ Touch Thermal Cycler (95°C/10 min, 40 cycles of 94°C/30s, and 55°C/1 min with a final stage at 98°C/10 min). Finally, all samples were analyzed on a Bio-Rad QX200 droplet reader.

## 3 Results

### 3.1 Detection of *PIK3CA* hotspot mutations in primary tumors

**Figure 1:**
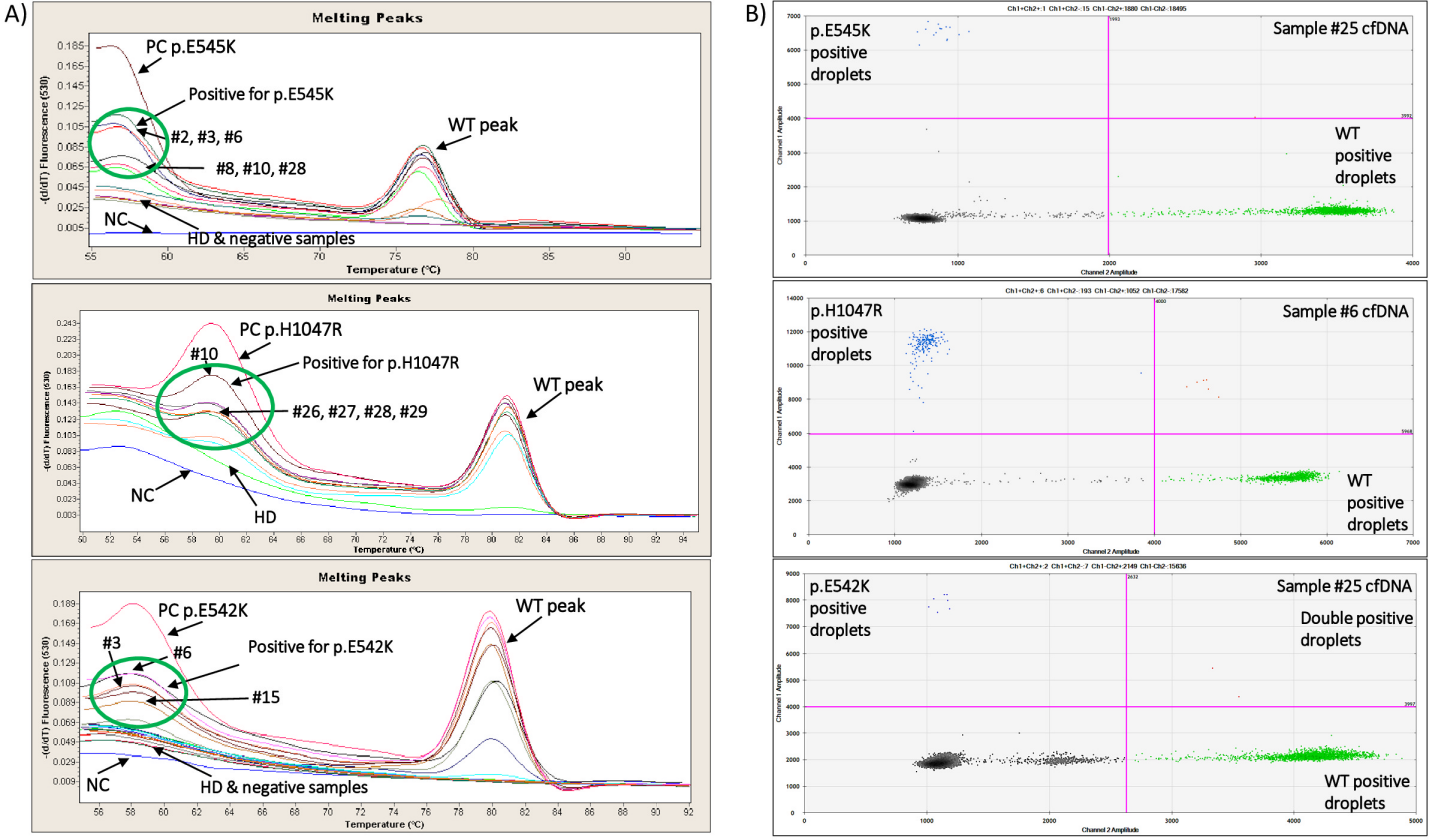
**(A)** Characteristic real-time PCR melting graphs for *PIK3CA* p.E545K, p.H1047R and p.E542K mutations of positive, negative and HD gDNA samples. **(B)** Characteristic ddPCR 2D dot-plots for *PIK3CA* p.E545K, p.H1047R and p.E542K mutations of 3 plasma cfDNA samples.

**Figure 2:**
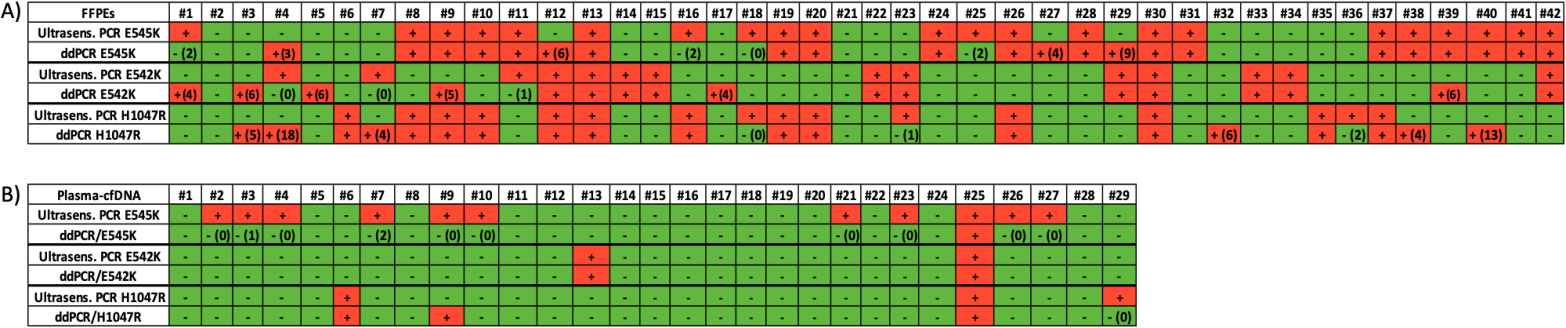
**(A)** Analysis of *PIK3CA* hotspot mutations in primary tumors (FFPEs gDNA). Direct comparison between the ultrasensitive real-time *PIK3CA* PCR assay with the ddPCR mutation test kit (Bio-Rad). Green: no mutation detected, red: mutation detected, ddPCR (#): number of droplets, **(B)** Analysis of *PIK3CA* hotspot mutations in plasma-cfDNA. Direct comparison between the ultrasensitive real-time *PIK3CA* PCR assay with the ddPCR mutation test kit (Bio-Rad). Green: no mutation detected, red: mutation detected, ddPCR (#): number of droplets.

The ultrasensitive real-time PCR *PIK3CA* assay was used to detect three *PIK3CA* hotspot mutations in 42 gDNA samples isolated from FFPEs. Characteristic real-time PCR melting graphs for these mutations are shown in **Figure 1A**. The p.E545K mutation was detected in 22/42(52.4%) samples, the p.E542K mutation in 14/42(33.3%) samples, and the p.H1047R mutation in 16/42(38.1%) samples (**Figure 2A**).

We further analyzed the same gDNAs (FFPEs) using the ddPCR *PIK3CA* mutation test kit (Bio-Rad) for the detection of p.E545K, p.E542K and p.H1047R mutations. Characteristic ddPCR 2D dot plots for these mutations are shown in **Figure 1B**. Using the ddPCR assay, we detected p.E545K mutation in 22/42(52.4%) samples (**Figure 2A**). The concordance between these two methodologies for p.E545K *PIK3CA* mutation was 81% (k= 0.618, p<0.001) (**Table 1**). Similarly, both methods detected p.E542K mutation in 14/42 (33.3%) samples. As shown in **Figure 2A**, for *PIK3CA* p.E542K mutation the ddPCR assay detected three more positive samples than the ultrasensitive real-time PCR *PIK3CA* assay, resulting in a concordance of 78.6% (k=0.542, p<0.001). As for the p.H1047R mutation, 16/42(38.1%) samples were found positive by both methods while 20/42 samples were found negative (**Figure 2A**) and discrepancies were detected in nine samples leading to a concordance of 78.6% with statistically significant difference (k=0.562, p<0.001), (**Table 1**).

**Table 1:** Direct comparison between the ultrasensitive real-time *PIK3CA* PCR assay and the ddPCR *PIK3CA* mutation test for *PIK3CA* p.E545K, p.E542K and p.H1047R mutations in 42 primary tumor samples (FFPEs).

		ddPCR *PIK3CA* Mutation Test kit			
**Ultrasensitive real-time *PIK3CA* PCR assay**	p.E545K		+	–	Total
		+	18	4	22
		–	4	16	20
		Total	22	20	42
		Concordance: **34/42 (81%), k= 0.618, p<0.001**			
	p.E542K		+	–	Total
		+	11	3	14
		–	6	22	28
		Total	17	25	42
		Concordance: **33/42 (78.6%), k= 0.542, p<0.001**			
	p.H1047R		+	–	Total
		+	13	3	16
		–	6	20	26
		Total	19	23	42
		Concordance: **33/42 (78.6%), k= 0.562, p<0.001**			

k= kappa coefficient.

### 3.2 Detection of *PIK3CA* hotspot mutations in plasma-cfDNA

Using the ultrasensitive real-time PCR assay, in plasma-cfDNA, at least one *PIK3CA* mutation was detected in 14/29(48.3%) samples analyzed. Specifically, the p.E545K mutation was detected in 11/29(38%) samples, and bothp.H1047R and p.E542K mutation in 2/29(6.9%) samples (**Figure 2B**).

We further performed a direct comparison of the ultrasensitive real-time PCR *PIK3CA* assay with ddPCR for *PIK3CA* mutation analysis using the same 29 plasma-cfDNA samples. Using ddPCR, the p.E545K mutation was detected in 1/29(3.5%) samples, the p.E542K mutation in 2/29(6.9%) samples, and the p.H1047R mutation in 3/29(10.5%) samples (**Figure 2B**).The results of p.H1047R and p.E542K revealed an excellent concordance for both mutations (**Table 2**). Among these 29 plasma-cfDNA samples, eleven were found to be positive for p.E545K mutation with the ultrasensitive real-time PCR *PIK3CA* assay while only one of them was found positive with the ddPCR *PIK3CA* mutation test. However, these ten samples that were found positive by the ultrasensitive real-time PCR and negative by ddPCR had a very low number of positive droplets (0-2 positive droplets). These discrepancies between ultrasensitive real-time PCR and ddPCR found in plasma-cfDNA samples could be due to sampling errors, that occur at very low analyte concentrations (Poisson distribution effect) especially in plasma-cfDNA (**Figure 2B**).

**Table 2:** Direct comparison between the ultrasensitive real-time *PIK3CA* PCR assay and the ddPCR *PIK3CA* Mutation Test for *PIK3CA* p.E545K, p.E542K and p.H1047R mutations in 29 plasma-cfDNA samples.

		ddPCR *PIK3CA* Mutation Test kit			
**Ultrasensitive real-time *PIK3CA* PCR assay**	p.E545K		+	–	Total
		+	1	10	11
		–	0	18	18
		Total	1	28	29
		Concordance: **19/29 (65.5%), k= 0.110, p=0.158**			
	p.E542K		+	–	Total
		+	2	0	2
		–	0	27	27
		Total	2	27	29
		Concordance: **29/29 (100%), k=1.000, p<0.001**			
	p.H1047R		+	–	Total
		+	2	1	3
		–	1	25	26
		Total	3	26	29
		Concordance: **27/29 (93.1%), k= 0.628, p=0.008**			

k= kappa coefficient.

### 3.3 Detection of *PIK3CA* hotspot mutations in paired CTC-derived gDNAs

**Figure 3:**
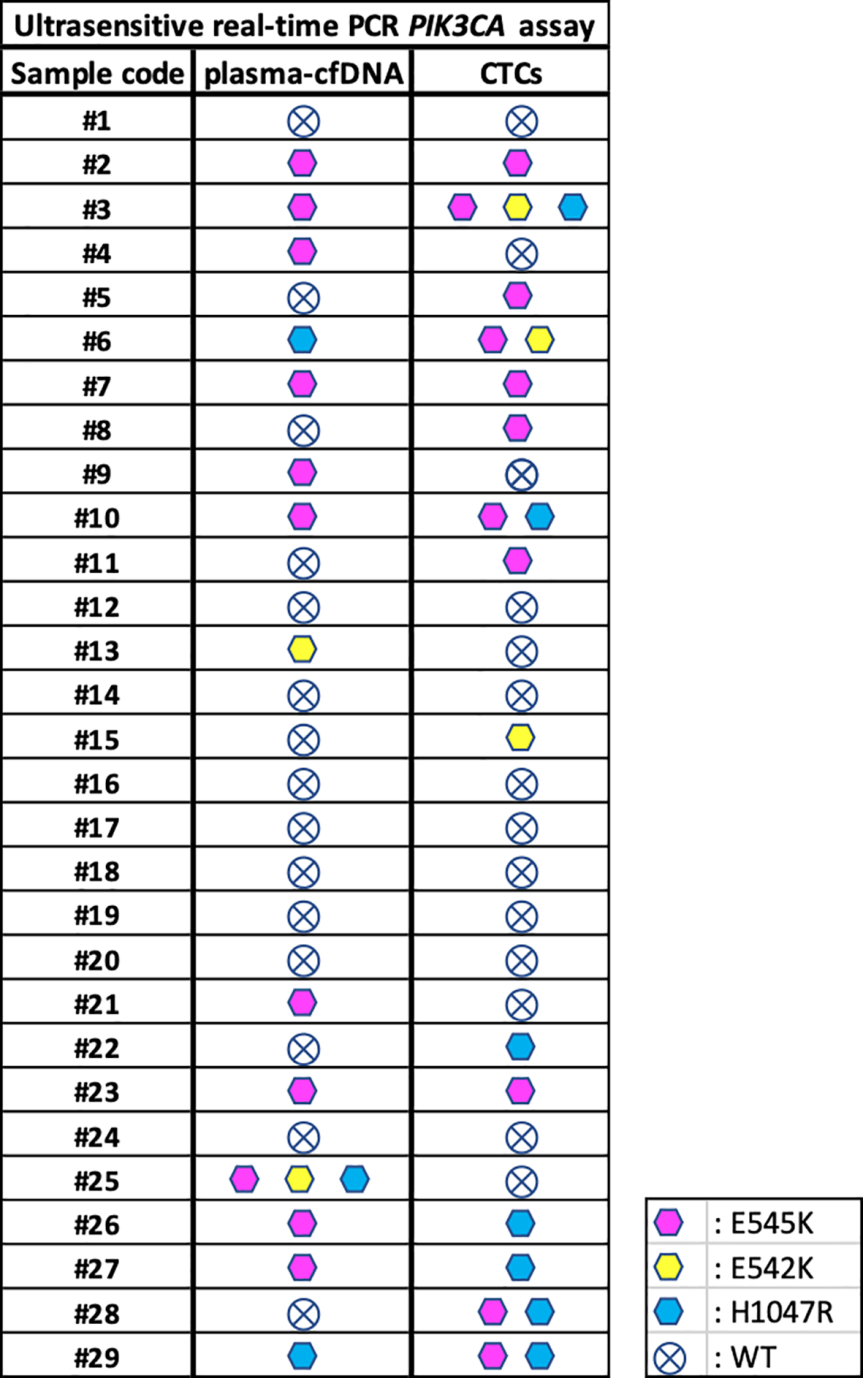
Direct comparison of *PIK3CA* mutations detected in plasma-cfDNA and paired CTC-derived gDNAs using the ultrasensitive real-time PCR assay.

Using the ultrasensitive real-time PCR *PIK3CA* assay we analyzed *PIK3CA* mutations in paired CTC-derived gDNAs. The p.E545K mutation was detected in 11/29 (38%) samples, the p.E542K mutation in 3/29 (10.3%) samples and the p.H1047R mutation in 7/29 (24.1%) samples. All results for 29 plasma-cfDNA and paired CTC-derived gDNAs are summarized in **Figure 3**. As can be seen, mutation percentages are slightly higher in CTCs than plasma-cfDNA as expected (38). From 29 patients with ER+/HER2- breast cancer, five were found to be positive for p.E545K mutation both in plasma-cfDNA and paired CTC gDNA and one for p.H1047R (**Figure 3**). Nine patients were negative for these *PIK3CA* mutations in both types of samples. Concordance rates between plasma-cfDNA and paired CTC-derived gDNA was 45.5% for *PIK3CA* p.E545K mutation. It has been observed that in some cases, the genetic mutations found in CTC-derived gDNAs do not match those detected in the plasma-cfDNA. Similarly, there are instances where plasma-cfDNA mutations are not present in CTC-derived gDNAs. Mutation rates for *PIK3CA* p.E542K and p.H1047R are very low as the number of positive samples were relatively low.

## 4 Discussion

Since *PIK3CA* mutations are present in multiple types of cancer, the reliable detection of these hotspot mutations is a highly important tool to guide treatment, especially since alpelicib is widely used in the treatment of ER+/HER2- metastatic breast cancer (18). Quick, reliable and inexpensive identification of *PIK3CA* mutations is crucial for determining which breast cancer patients will benefit from these specific treatments (48). The presence of *PIK3CA* mutations in exon 9 was found to be associated with a more favorable outcome in some medical conditions. On the other hand, the presence of *PIK3CA* mutations in exon 20, was found to be linked to a relatively poor prognosis, indicating a higher likelihood of negative outcomes (49, 50).

In this study we directly compared the efficacy of a relatively inexpensive ultrasensitive real-time PCR with the now well-established and highly sensitive technology of ddPCR for the detection of the three most common hotspot mutations of *PIK3CA*, in exons 9 and 20, that are all of clinical importance in various types of cancer. According to our results, this simple and ultrasensitive real-time PCR assay gives similar results to ddPCR, can be performed in labs where digital PCR instrumentation is not available and provides accurate and specific detection of *PIK3CA* hotspot mutations in liquid biopsy samples.

The high positivity for *PIK3CA* mutations in primary tumors FFPE samples, detected by this assay is in agreement with previously reported studies (35, 47). In FFPEs our direct comparison study between the ultrasensitive real-time PCR assay with ddPCR for p.E545K, revealed a relatively good agreement, while the detection of p.E542K and p.H1047R was also in good agreement between the two assays. Using ddPCR three more samples were detected as positive for the p.E542K and p.H1047R in FFPEs. When classic real-time PCR was directly compared with ddPCR for the detection of *PIK3CA* mutations in FFPEs of Head and Neck Squamous Cell Carcinoma patients, it was reported that ddPCR was superior in terms of sensitivity in the *PIK3CA* mutation assessment in FFPE samples (51).

In plasma-cfDNA, the rates of *PIK3CA* positiveness usually reported are relatively low. Using the ultrasensitive real-time PCR *PIK3CA* assay ten plasma-cfDNA samples were found positive for the p.E545K mutation while they were found negative by ddPCR. These ten samples that are reported as negative using ddPCR for the *PIK3CA* p.E545K mutation were found to have between 0 to 2 droplets, indicating the absence of positive events, since the p.E545K positive copies detected are extremely low to be called as positive events. On the other hand, two plasma-cfDNA samples were found positive for the *PIK3CA*p.E542K mutation with both assays leading to complete agreement. Moreover, two plasma-cfDNA samples were found positive for p.H1047R mutation by the ultrasensitive real-time PCR *PIK3CA* assay, while by ddPCR p.H1047R mutation was detected in three plasma-cfDNA, revealing a strong agreement for this mutation too.

Analysis of *PIK3CA* mutations in CTC-derived gDNAs showed a higher percentage of *PIK3CA* hotspot mutations than in plasma-cfDNA, especially for p.H1047R. Moreover, we observed that when we compared mutations identified in CTCs and plasma-cfDNA, *PIK3CA* mutations were detected in CTCs but were not detectable in plasma-cfDNA in some peripheral blood samples, whereas in other blood samples, *PIK3CA* mutations were detected in plasma-cfDNA but were not detected in CTCs. Our results indicate that CTC and plasma-cfDNA give complementary information, especially at very low analyte concentrations, and that evaluating both CTCs and plasma-cfDNA analysis is necessary to ensure optimal disease surveillance and appropriate treatment selection in the clinical setting. We have recently shown that comprehensive liquid biopsy analysis including the analysis of *PIK3CA* mutations in CTCs and ctDNA is a very informative tool for the early detection of minimal residual disease in breast cancer (52). Based on a relatively small number of patients another study has come to the conclusion that combined analysis of CTCs and ctDNA may offer a new approach for monitoring of disease progression and to direct therapy in patients with advanced MBC, at a time when they are coming towards the end of other treatment options (53). Molecular analysis, performed both in CTCs and plasma-cfDNA, provides results that reflect certain subpopulations of the primary tumor as well as cells forming metastases and should be considered an important form to monitor the development of the disease and its status over time, particularly for its clinical impact in guiding drug selection. We have already shown in a previous study that the prevalence of detectable mutations of *PIK3CA* was higher in CTC-derived DNA than in the corresponding plasma-cfDNA (54).

ddPCR is considered as the next generation of PCR technology, since it offers absolute quantification of nucleic acid target sequences without the need for external calibration curves. However digital PCR is still very expensive in terms of both instrumentation and reagents, and is still not available in most molecular diagnostic labs worldwide. Real-time PCR still offers numerous benefits, including established protocols, well-known data analysis techniques, and availability of necessary instrumentation worldwide. Additionally, it boasts a significant low experiment cost, high sample throughputs, less consumables and a wide dynamic range for detection. Real-time PCR in combination with melting curve analysis is highly effective for mutational screening. This methodology is highly sensitive and provides quick and accurate results, making it a suitable option for cases where absolute quantification is not necessary. In our hands ultrasensitive real-time PCR for *PIK3CA* mutations showed similar sensitivity to ddPCR, however the limited number of clinical samples and the relatively small number of healthy controls analyzed, restricts the conclusions made. In CTC-derived gDNA and paired plasma-cfDNA, *PIK3CA* mutations detected were not identical, revealing that CTC and plasma-cfDNA give complementary information.

In conclusion, molecular diagnostic applications for *PIK3CA* mutation screening in liquid biopsy samples that require high sensitivity and precision can greatly benefit from the presented reliable, low-cost and high-throughput ultrasensitive real-time PCR assay.

## Data Availability

The raw data supporting the conclusions of this article will be made available by the authors, without undue reservation.
